# Evaluation of Radiation dosimetry of ^99m^Tc-HYNIC-PSMA and imaging in prostate cancer

**DOI:** 10.1038/s41598-020-61129-5

**Published:** 2020-03-06

**Authors:** Jianping Zhang, Jiangang Zhang, Xiaoping Xu, Linjun Lu, Silong Hu, Chang Liu, Jingyi Cheng, Shaoli Song, Yingjian Zhang, L. Q. Shi

**Affiliations:** 1 Key Laboratory of Nuclear Physics and Ion-Beam Application (MOE), Fudan University, No. 220, Handan Road, Yangpu District, Shanghai, 200433 China; 2 Institute of Modern Physics, Fudan University, No. 220, Handan Road, Yangpu District, Shanghai, 200433 China; 3 Department of Nuclear Medicine, Fudan University Shanghai Cancer Center, No. 270, Dong’an Road, Xuhui District, Shanghai, 200032 China; 4 Department of Oncology, Shanghai Medical College, Fudan University, No. 130, Dong’an Road, Xuhui District, Shanghai, 200032 China; 5 Center for Biomedical Imaging, Fudan University, No. 270, Dong’an Road, Shanghai, 200032 China; 6 Shanghai Engineering Research Center for Molecular Imaging Probes, No. 270, Dong’an Road, Xuhui District, Shanghai, 200032 China; 7 Department of Nuclear Medicine, Shanghai Proton and Heavy Ion Center, Fudan University Cancer Hospital, No. 4365, Kangxin Road, Pudong New District, Shanghai, 201315 China; 8 Shanghai Engineering Research Center of Proton and Heavy Ion Radiation Therapy, No. 4365, Kangxin Road, Pudong New District, Shanghai, 201315 China

**Keywords:** Cancer imaging, Cancer imaging

## Abstract

This study aims to evaluate the radiation dosimetry of a new technetium-99m‒labelled small-molecule inhibitor of prostate-specific membrane antigen (HYNIC-Glu-Urea-A, ^99m^Tc-HYNIC-PSMA) and its feasibility as a tumor-imaging agent in prostate cancer (PCa) patients. A total of 15 PCa patients were enrolled in this study. For the dosimetry study, 5 PCa patients received whole-body planar scans at 0.5 h, 1 h, 2 h, 4 h and 8 h after ^99m^Tc-HYNIC-PSMA injection. The Dosimetry Toolkit (GE, Milwaukee) was used to process the data and segment the organs in the SPECT/CT images, which were then projected onto planar images. The organ-specific absorbed doses, total-body absorbed doses and ^99m^Tc-HYNIC-PSMA effective doses of patients were calculated using OLINDA/EXM 1.1 software. Whole-body SPECT/CT images were also acquired from additional 10 prostate patients to investigate the feasibility of ^99m^Tc-HYNIC-PSMA for imaging tumors by calculating the ratio of tumor-to-background tracer uptake at 2 h after 740 MBq administration. The total-body absorbed dose was 1.54E-03 ± 2.43E-04 mGy/MBq, and the effective dose was 3.72E-03 ± 4.5E-04 mSv/MBq. Compared to published studies of other similar PSMA tracers and ^99m^Tc-targeted conventional tracers, the absorbed doses of ^99m^Tc-HYNIC-PSMA in all organs showed that it could be used safely in the human body. In addition, ^99m^Tc-HYNIC-PSMA showed high tracer uptake (with a tumor-to-background ratio of 9.42 ± 2.62) in the malignant lesions of PCa patients, making it a promising radiopharmaceutical imaging method for site-specific management of PCa.

## Introduction

Prostate cancer (PCa) is one of the most common malignant tumors diagnosed in middle-aged and older male patients and it is now the second leading cause of cancer deaths in males^1^. Various studies have found that prostate-specific membrane antigen (PSMA), a metallopeptidase, is highly overexpressed on the surfaces of PCa cells^2^, making it a valuable research object in the field of molecular imaging and target therapy for PCa^3–8^.

Due to the exclusively high expression of PSMA by PCa, radionuclide-labelled PSMA small-molecule inhibitors have been reported as a promising radiopharmaceuticals for the clinical application to molecular imaging for PCa diagnosis^9,10^, one of which is HYNIC-Glu-Urea-A (^99m^Tc-HYNIC-PSMA). Its targeting property has been evaluated in both *in vitro* and *in vivo* using PCa models (PC-3 for PSMA^−^ and LNCaP for PSMA^+^) which revealed a clear difference between the two models^11^. This result suggests that ^99m^Tc-HYNIC-PSMA is a promising SPECT/CT imaging agent for the PSMA^+^ PCa. Although ^68^Ga-labelled PSMA tracers can clearly reveal tumor lesions by PET/CT^10^, in terms of the expense as well as the facility availability, ^99m^Tc-labelled PSMA tracers possess a greater potential for a more widespread clinical application, especially in small- and medium-sized medical institutions.

The calculation of radiation dosimetry is crucial in evaluating the safety of radiopharmaceuticals. In this study, using the method established by Committee on Medical Internal Radiation Dose (MIRD) with the help of the Dosimetry Toolkit (DTK, GE, Milwaukee)^12,13^, we calculated the radiation dosimetry of ^99m^Tc-HYNIC-PSMA for 5 PCa patients. Furthermore, the imaging feasibility of ^99m^Tc-HYNIC-PSMA was also evaluated in 10 PCa patients.

## Results

### Biodistribution and time-ID% curve of each source organ

**Figure 1:**
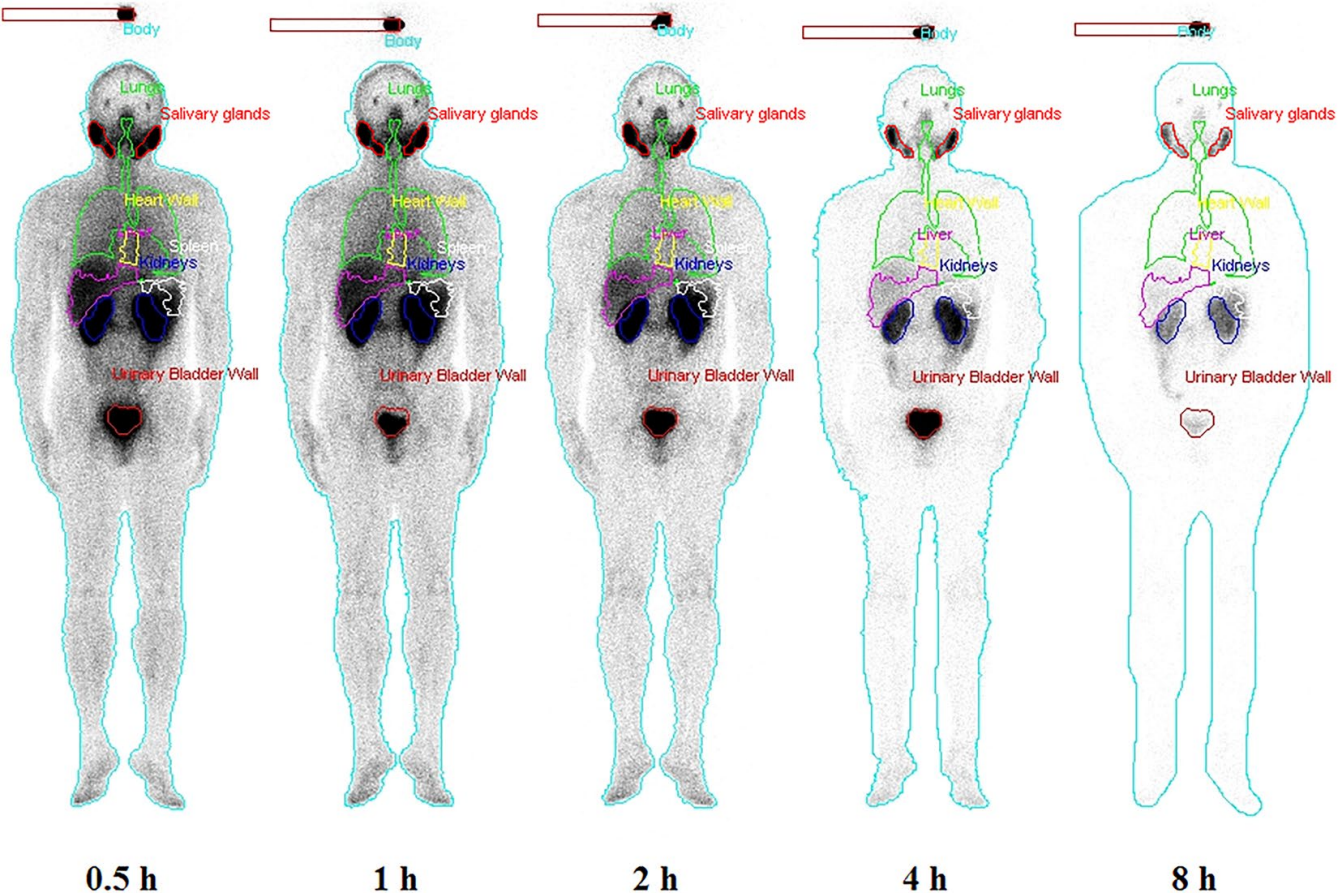
Whole-body images and segmented source organs of a subject from 0.5 h to 8 h after ^99m^Tc-HYNIC-PSMA administration (740 MBq).

**Figure 2:**
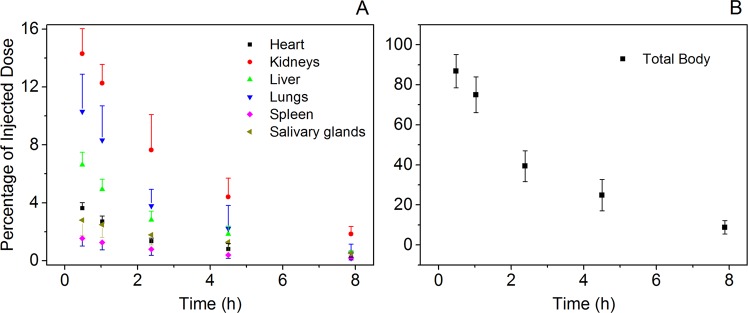
Mean percentage of injected dose (ID%) as a percentage of the initial total dose for source organs and for the total body determined from the 5 enrolled subject ^99m^Tc-HYNIC-PSMA SPECT scans, as a function of time after injection. (**A**) For kidneys, lungs, liver, heart, salivary glands and spleen and (**B**) for the total body.

The radioactivity in organs was determined according to the biodistribution in humans (Fig. 1). High tracer uptake was observed in the kidneys, bladder, parotid gland and salivary gland. The ragged ROIs observed for the liver and spleen were caused by the automatic overlapping correction by the DTK in hybrid mode. This correction automatically removed overlapping components, such as the liver and right lung. The activity distributions of the removed ROIs were assumed to be uniform and were substituted by the mean activity concentration estimated from the remaining organ parts. Figure 2A showed the decay-corrected time-activity scatters for the human liver, heart, lungs, spleen, salivary glands and kidneys after ^99m^Tc-HYNIC-PSMA administration, and the results of which were consistent with the biodistribution images in this study (Fig. 1). Compared to other organs, the kidneys displayed the highest uptake of ^99m^Tc-HYNIC-PSMA throughout the test while the spleen and heart had relatively low levels of uptake. The highest *%ID* values in the kidneys, lungs, liver, heart, salivary glands and spleen were found at 0.5 h where the values (representing the mean ± SD for n = 5 patients) are 14.3 ± 1.72, 10.31 ± 2.57, 6.63 ± 0.88, 3.63 ± 0.38, 2.80 ± 1.07 and 1.54 ± 0.52, respectively. The total body time–*ID%* scatter (Fig. 2B) illustrated a quick excretion of the tracer, since only approximately 10% was remained after 8 h.

### Time integrated activity coefficients (TIACs) in source organs and bladder

The *%ID* curve of ^99m^Tc-HYNIC-PSMA in the bladder was fitted with a double exponential function as shown in Eqn. (4) in the methods section. The half-life value of the exponential function (the period of time it takes for the exponential function to decrease by half) was 1.15 ± 0.75 h for the fast component, which had a fraction of 58.23% ± 6.83%, while the slow component half-life was 3.06 ± 1.49 h, with a fraction of 32.98% ± 4.83%. According to a voiding interval of 2 h, the TIAC of the urinary bladder content was 8.42E-01 ± 2.00E-01 MBq·h/MBq.

Table 1 lists the TIAC of each source organ (kidneys, lungs, liver, heart, salivary glands and spleen), the bladder and the remainder of the body. The results showed that the bladder had the largest TIAC, followed by the kidneys. The TIACs in the lungs, liver, heart, salivary glands and spleen were considerably smaller.

**Table 1:** Time integrated activity coefficients (TIACs) of ^99m^Tc-HYNIC-PSMA for source organs and remainder of body. (Data are mean ± SD; n = 5).

Source organ	TIAC (MBq·h/MBq)
Heart	9.82E-02 ± 2.44E-02
Kidneys	5.42E-01 ± 1.23E-01
Liver	2.06E-01 ± 3.60E-02
Lungs	3.29E-01 ± 1.39E-01
Salivary glands	9.27E-02 ± 3.19E-02
Spleen	5.29E-02 ± 2.67E-02
Bladder	8.42E-01 ± 2.00E-01
Remainder of body	8.25E-01 ± 4.63E-01

### Radiation dosimetry

Table 2 lists the organ-absorbed doses and the effective doses. The lungs, salivary glands, spleen, kidneys and bladder had higher absorbed doses than the breasts, thyroid, skin and brain. The radiation-sensitive organs, including osteogenic cells, thymus and red bone marrow, showed low absorbed doses that ranged from 1.21E-03 ± 4.40E-04 to 2.38E-03 ± 7.75E-04 mGy/MBq. The total-body absorbed dose was 1.54E-03 ± 2.43E-04 mGy/MBq, and the effective dose was 3.72E-03 ± 4.50E-04 mSv/MBq.

**Table 2:** Subjects absorbed dose and effective dose of ^99m^Tc-HYNIC-PSMA.

Target organ	Absorbed dose (mGy/MBq)
Adrenals	2.84E-03 ± 3.78E-04
Brain	4.12E-04 ± 2.34E-04
Breasts	8.04E-04 ± 2.97E-04
Gallbladder	2.31E-03 ± 3.66E-04
Lower large intestine wall	2.60E-03 ± 3.87E-04
Small intestine wall	1.88E-03 ± 1.99E-04
Stomach wall	1.58E-03 ± 2.75E-04
Upper large intestine wall	1.71E-03 ± 2.20E-04
Heart wall	3.87E-03 ± 1.53E-03
Kidneys	2.87E-02 ± 1.53E-03
Liver	3.76E-03 ± 6.06E-04
Lungs	5.41E-03 ± 2.10E-03
Muscle	1.28E-03 ± 2.05E-04
Pancreas	2.49E-03 ± 3.16E-04
Red marrow	1.32E-03 ± 2.43E-04
Osteogenic cells	2.38E-03 ± 7.75E-04
Skin	6.00E-04 ± 1.61E-04
Spleen	6.68E-03 ± 2.20E-03
Salivary glands	1.28E-02 ± 4.25E-03
Testes	1.65E-03 ± 2.56E-04
Thymus	1.21E-03 ± 4.40E-04
Thyroid	6.20E-04 ± 3.10E-04
Urinary bladder wall	3.53E-02 ± 9.01E-03
Total body	1.54E-03 ± 2.43E-04
Effective dose (mSv/MBq)	3.72E-03 ± 4.50E-04

(Data are mean ± SD; n = 5).

#### Evaluation of malignant uptake

**Figure 3:**
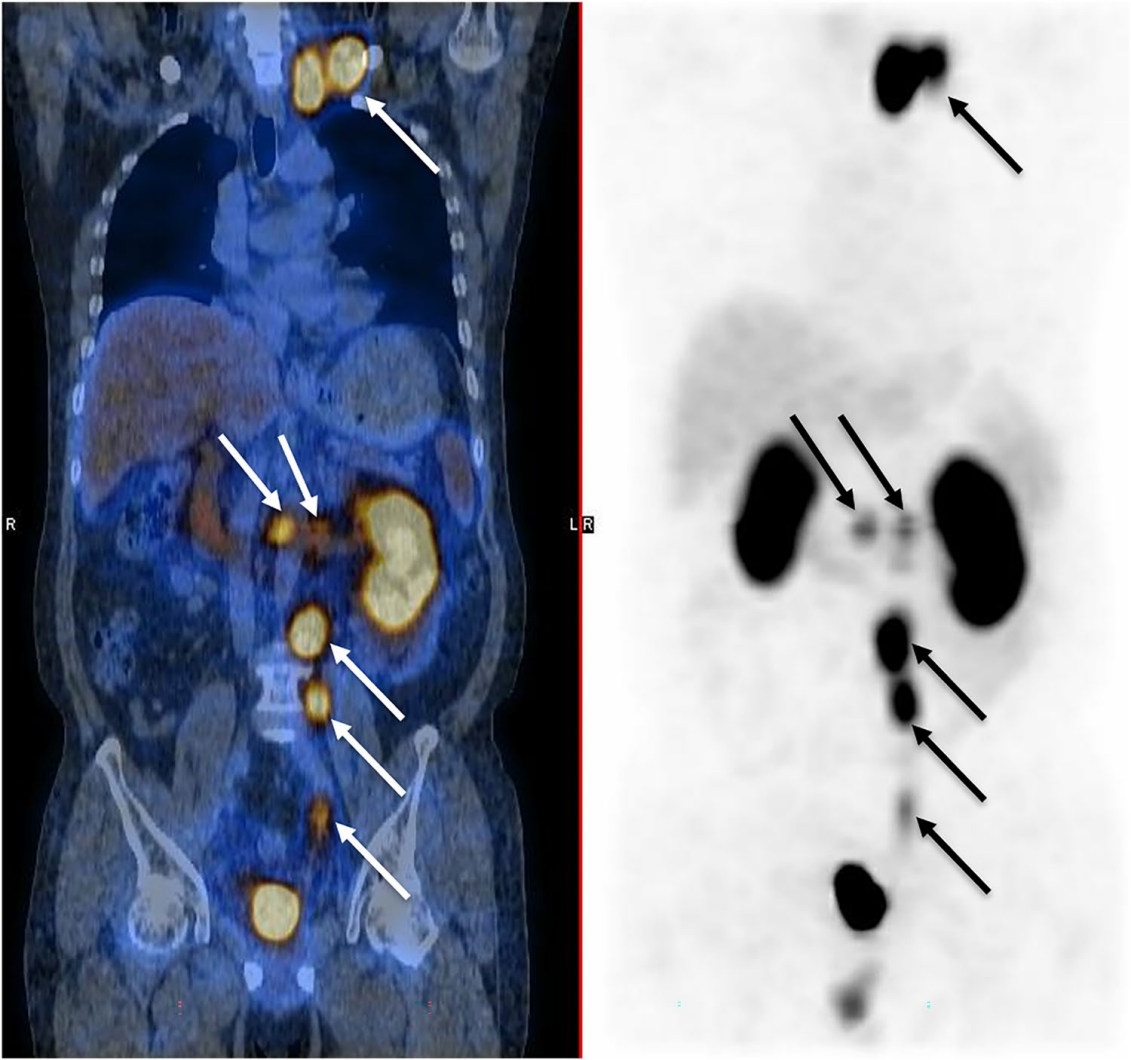
Maximum intensity projection (right) and fused (left) ^99m^Tc-HYNIC-PSMA SPECT/CT images of a 59-year-old patient, who previously received a radical prostatectomy, 2 h after administration of 740 MBq dose. Multiple lymph node metastases were detected (arrowed) and the left supraclavicular lymph node has a maximum tumor-to-background ratio of 9.2.

For the tracer imaging feasibility study in 10 patients, a high tumor-to-background ratio ranging from 4.7 to 15.3 (average 9.42 ± 2.62 at 2 h after administration, Table 3) for ^99m^Tc-HYNIC-PSMA was observed in malignant lesions. Figure 3 demonstrates the maximum intensity projection and fused SPECT/CT images of a post-prostatectomy 59-year-old patient at 2 h after administration of 740 MBq ^99m^Tc-HYNIC-PSMA. Multiple lymph nodes metastases were observed in both images and the left supraclavicular lymph node had a maximum tumor-to-background ratio of 9.2.

**Table 3:** Accumulation of ^99m^Tc-HYNIC-PSMA in tumors of 10 prostate cancer patients expressed as tumor-to-background ratio at 2 h after administration.

Patient	Age	Histopathology report	Gleason	Status	Imaging time-point PSA value (ng/ml)	Lesion location	Tumor-to-background ratio
1	72	Acinar adenocarcinoma	4 + 4	Pre-treatment	17.21	prostate	11.2
2	65	Acinar adenocarcinoma	5 + 4	Biochemical relapse	2.12	Retroperitoneal lymph node	7.6
3	77	Duct adenocarcinoma	5 + 5	Biochemical relapse	0.56	No. 3 lumbar spine	8.9
4	52	Acinar adenocarcinoma	4 + 3	Pre-treatment	10.78	prostate	9.2
5	66	Acinar adenocarcinoma	4 + 4	Pre-treatment	25.29	prostate	8.5
6	77	Duct adenocarcinoma	4 + 4	Pre-treatment	32.23	prostate	15.3
7	73	Acinar adenocarcinoma	4 + 5	Biochemical relapse	1.02	prostatectomy bed	4.7
8	59	Acinar adenocarcinoma	5 + 4	Biochemical relapse	2.21	Left supraclavicular lymph node	9.2
9	68	Acinar adenocarcinoma	4 + 3	CRPC	27.88	Left tibia	8.4
10	70	Acinar adenocarcinoma	4 + 5	CRPC	11.66	Right para-vascular lymph node	11.2

CRPC: Castration-Resistant Prostate Cancer.

## Discussion

To date, several ^99m^Tc-labelled PSMA inhibitors have been developed for PCa detection, including ^99m^Tc-MIP-1404^9^,^99m^Tc-MIP-1405^9^, ^99m^Tc-PSMA-I&S^14^ and ^99m^Tc-EDDA/HYNIC-iPSMA^15^. Compared with other imaging techniques, such as CT and MRI, SPECT/CT using ^99m^Tc-labelled PSMA has demonstrated great potential for detecting PCa metastasis and guiding the treatment of targeted lesions, thus benefiting patients^7,10^.

The present study conducted a clinical safety evaluation of ^99m^Tc-HYNIC-PSMA according to a method that we developed^16^. Our results suggest that ^99m^Tc-HYNIC-PSMA was excreted mainly through the urinary system and that its absorbed doses in organs, including the brain and heart, were low. The kidneys showed the highest absorbed dose, which had a value of 2.87E-02 ± 1.53E-03 mGy/MBq. In other organs, including the red bone marrow, thyroid and adrenal gland, the absorbed doses were lower. The effective dose of ^99m^Tc-HYNIC-PSMA was 3.72E-03 ± 4.50E-04 mSv/MBq, so it is 2.75 ± 0.33 mSv when the administered activity was 740 MBq. This effective dose value is similar to that of ^99m^Tc-EDDA/HYNIC-iPSMA^15^ (3.73E-03 mSv/MBq) but lower than that of ^99m^Tc-MIP-1404^9^ (8.8E-03 mSv/MBq) and ^99m^Tc-MIP-1405^9^ (7.9E-03 mSv/MBq). The total effective dose in the body is also much lower than that of ^68^Ga-labelled and ^18^F-labelled PSMA-targeted tracers, including ^68^Ga-PSMA-11^17^ (2.36E-02 mSv/MBq), ^68^Ga-PSMA-617^18^ (2.1E-02 mSv/MBq), ^18^F-PSMA-1007^19^ (2.2E-02 mSv/MBq) and ^18^F-DCFPyL^20^ (1.39E-02 mSv/MBq). This is because ^68^Ga and ^18^F have a dual-photon property and emit gamma radiation with higher energy than ^99m^Tc. Furthermore, the effective dose of ^99m^Tc-HYNIC-PSMA is also lower than that of conventional ^99m^Tc-labelled radiopharmaceuticals used in SPECT/CT scans, such as MDP (5.68E-03 mSv/MBq)^21^ and MIBI (7.83E-03 mSv/MBq)^21^. Of note, because the main excretory pathways for the tracer are the kidneys and bladder, which is in agreement with the results shown in our current study, we suggest that patients drink plenty of water or take diuretics after the scan to reduce the absorbed dose.

The Dosimetry Toolkit supports 3 different scenarios, including multiple whole-body SPECT/CT scenarios, multiple whole-body planar scenarios and a hybrid planar-SPECT/CT scenario. In order to avoid the long scan time required by multiple whole-body SPECT imaging, which could cause tracer metabolic difference between segments for each scan, the hybrid imaging scenario was chosen. Conventionally, for organ segmentation, it is very difficult to manually utilize information from 2D planar images, particularly for those regions with low tracer uptake. Unlike the conventional technique, this computer-assisted semiautomatic method provides a more consistent and convenient method for image processing.

**Figure 4:**
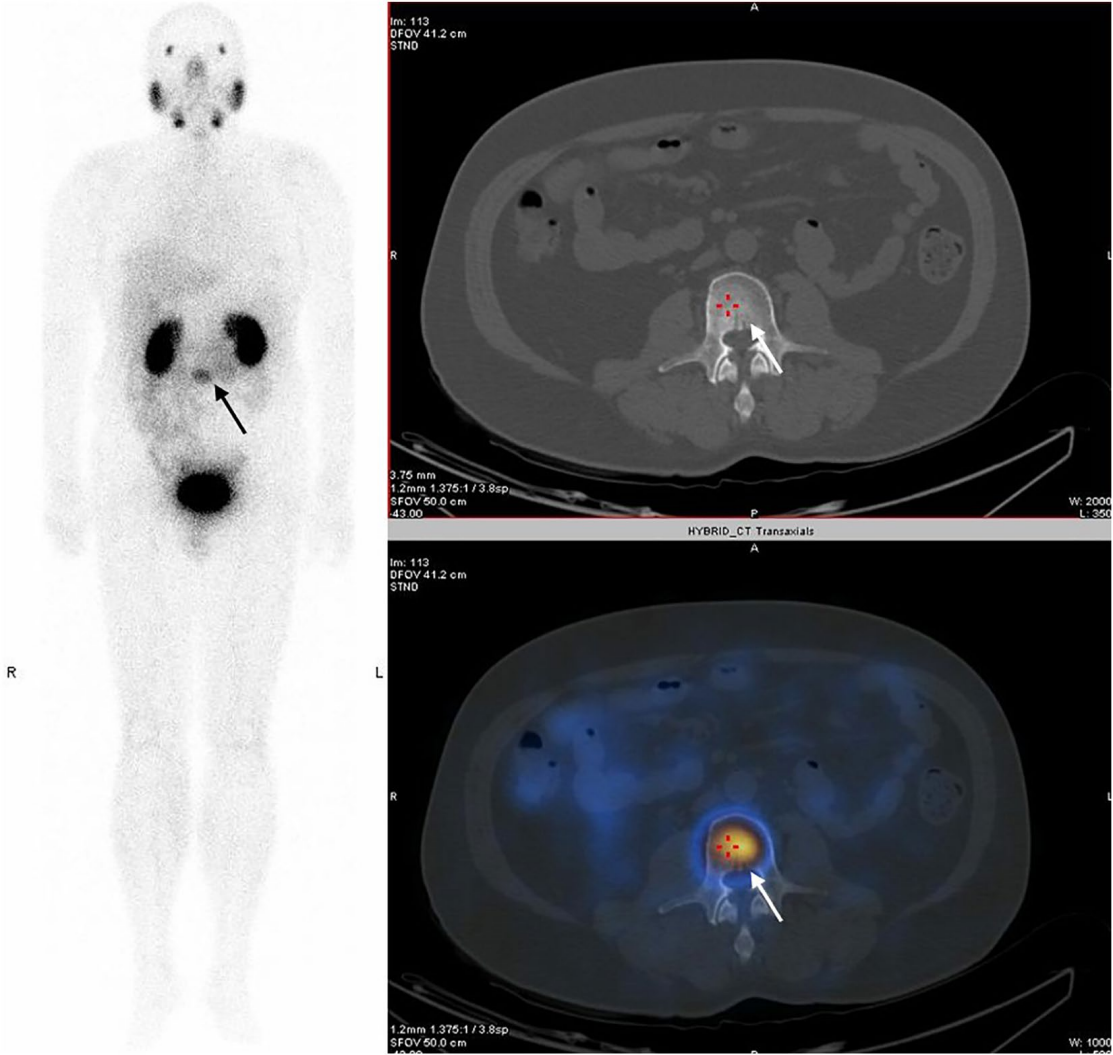
Maximum intensity projection (left), CT (top right) and fused (bottom right) ^99m^Tc-HYNIC-PSMA SPECT/CT images of a 77-year-old patient with a PSA of only 0.56. A L3 vertebral metastasis was detected (arrowed) in fused image, but no perceptible lesion was found on CT anatomical imaging.

For the tracer imaging feasibility study, Table 3 showed that the ^99m^Tc-HYNIC-PSMA was able to be accumulated at primary lesion sites and metastatic lesion sites (bone, soft tissue and lymph nodes). The average tumor-to-background ratio for ^99m^Tc-HYNIC-PSMA at 2 h was 9.42 ± 2.62, which is moderately higher than that observed for ^99m^Tc-MIPs (range 3.8–6.2)^9^. When the PSA is very low (Patient #3 from Table 3 as an example, with PSA of only 0.56), the ^99m^Tc-HYNIC-PSMA image can detect the uptake of the malignant lesion, while the anatomical imaging of the patient did not show any corresponding lesions (Fig. 4).

The main limitation of this study is the small sample size (5 patients were included in the dosimetry investigation and 10 patients were used for the validation of the usefulness of ^99m^Tc-HYNIC-PSMA), but the small standard deviation of the effective dose suggests that our analysis is reproducible for the dose calculation of ^99m^Tc-HYNIC-PSMA. Another limitation is due to the imaging protocol which has an experimental period of 8 h for each patient, so it is very difficult to guarantee a consistent scanning position, which would likely lead to tiny organ shifts. Additionally, there are also limitations for the hypotheses which were proposed based on the dose estimation process. Firstly, according to the method established by MIRD, radioactivity is supposed to be evenly distributed throughout the body instantly after administration. Secondly, any unmeasured radioactivity is assigned to the other organs in the body.

## Conclusions

In this study, ^99m^Tc-HYNIC-PSMA was shown a high specific uptake (with a tumor-to-background ratio of 9.42 ± 2.62) in the malignant lesions of PCa patients, and it was found the urinary system to be its main excretory pathway. Our dosimetry study also showed that, at the routine clinical dose (740 MBq), the effective dose of ^99m^Tc-HYNIC-PSMA was 2.75 ± 0.33 mSv (range: 2.27–3.19 mSv) which is similar to the effective doses from other PSMA inhibitors, such as ^99m^Tc-EDDA/HYNIC-iPSMA published in the literature, so it indicates that could be a safe SPECT tracer.

## Materials and methods

All studies were approved by the Fudan University Shanghai Cancer Center ethics committee, and all the procedures in the studies involving human participants were performed in accordance with the ethical standards of the institution. For the dosimetry study, each subject signed a written informed consent form prior to participating in the ^99m^Tc-HYNIC-PSMA dosimetry study. For the imaging feasibility study, a retrospective review on patients was conducted, and its results did not influence further therapeutic decision-making, so the ethics committee approved that informal consent was not required.

### Subjects

From May to June 2018, 5 PCa patients (mean age ± SD, 60 ± 6 years; age range, 48–68 years) underwent ^99m^Tc-HYNIC-PSMA single-photon emission computed tomography/computed tomography (SPECT/CT) scanning. Unlike conventional PCa patients, in order to mimic the conditions in healthy subjects, the five patients selected in this study did not show obvious tracer accumulation in tumor lesions or metastatic sites in the SPECT/CT scans. The biodistribution of radiopharmaceuticals throughout their bodies were similar to that in healthy adult males. Two patients had made only their first-visit, so were untreated, while the other three had received a radical prostatectomy. One of these three patients had experienced a biochemical recurrence. None of the patients received any radiation therapy or chemotherapy treatment. For the imaging feasibility study using ^99m^Tc-HYNIC-PSMA, which went from January to May 2019, 10 patients aged 52 to 77 years (mean age ± SD, 68 ± 8 years) were enrolled, and all patients were histologically diagnosed with PCa. In addition, all lesions detected in the imaging feasibility study were confirmed by biopsy or clinical follow-up.

### ^99m^Tc-HYNIC-PSMA synthesis and administration

^99m^Tc-HYNIC-PSMA was synthesized using a method developed by our team^11^. In this method, 10 μg of HYNIC-Glu-Urea-A, 0.5 mL of EDDA, 0.5 mL of Tricine and 25 μg of SnCl_2_ solution were allowed to react with 1110–4440 MBq of Na^99m^TcO_4_. The reaction was carried out in a water bath that was boiled for 10 min and then cooled to room temperature. The results showed that a labelling yield of more than 99% was achieved, and no additional purification was needed. In addition, endotoxin tests and retrospective analysis of the bacterial cultures all showed negative results. All patients were asked to void their bladders before receiving intravenous injections of ^99m^Tc-HYNIC-PSMA (mean activity ± SD, 740 ± 74 MBq).

### Image acquisition

Whole-body planar images (anterior, ANT; posterior, POST) were collected at 0.5 h, 1 h, 2 h, 4 h and 8 h after the injections, respectively, to estimate the dosimetry of ^99m^Tc-HYNIC-PSMA. A ^99m^Tc-HYNIC-PSMA reference source with a known activity (37 MBq) was prepared and placed 10 cm above each patient’s vertex during each scan for the conversion of the count per minute (cpm) to activity. Data were acquired using a dual-detector SPECT/CT instrument (Discovery NM/CT 670, GE, Milwaukee) with low-energy, high-resolution, parallel-hole collimators. The patients were asked to void their bladders before each scan made at 2 h, 4 h and 8 h respectively. For the whole-body planar images, the scanning parameters were chosen as follows: the main energy window was 140 keV ± 10%, the scatter energy window was 120 keV ± 5%, the matrix size was 256 × 1024 and the scan speed was 15 cm/min. The SPECT/CT scan was performed immediately following the whole-body planar image scan performed after 2 h; the patients’ positions remaining unchanged. Scans were conducted from each patient’s apex pulmonis to his pubic symphysis and included the chest, abdominal cavity and pelvic cavity. SPECT scans were first performed using the following acquisition parameters: the matrix size, 128 × 128; Zoom, 1; acquisition was over 360 degrees in 6 degree increments taking a total of 30 minutes. Then, conventional low-dose CT was conducted.

For the study involving the validation of the usefulness of ^99m^Tc-HYNIC-PSMA in 10 PCa patients, SPECT/CT images were acquired at 2 h after radiopharmaceutical administration. The SPECT/CT scan protocol was the same as that stated above. ROIs were drawn around tumors, and for the background, a circular ROI with a diameter of 2 cm was drawn within the obturator muscle.

### Image processing

The ANT and POST images were processed into geometric mean (GM) images with scatter correction. The collected SPECT data were reconstructed using an iterative algorithm with resolution recovery, attenuation, decay and scatter corrections. The volumes of interest (VOIs) were delineated both manually in the CT imaging (for low uptake areas and overlapping organs such as lungs, liver and spleen) and automatically in the SPECT imaging (for high uptake areas and organs or regions that did not overlap such as the kidneys, salivary glands, heart and the total body) by a medical physicist and a radiologist using the Dosimetry Toolkit. The whole-body GM images from other time points were automatically registered to the GM image collected at 2 h. Then, the organ segmentations VOIs were projected onto the GM images.

### TIACs and absorbed dose calculations

At each time point, the percentage of injected dose (*ID%*) of each source organ was assessed according to the following formula:

1 $${\rm{ \% }}I{D}_{{\rm{s}}{\rm{o}}{\rm{u}}{\rm{r}}{\rm{c}}{\rm{e}}{\rm{o}}{\rm{r}}{\rm{g}}{\rm{a}}{\rm{n}}}\,(t)=\frac{{A}_{{\rm{s}}{\rm{o}}{\rm{u}}{\rm{r}}{\rm{c}}{\rm{e}}{\rm{o}}{\rm{r}}{\rm{g}}{\rm{a}}{\rm{n}}}\,(t)}{{A}_{{\rm{i}}{\rm{n}}{\rm{j}}{\rm{e}}{\rm{c}}{\rm{t}}{\rm{e}}{\rm{d}}{\rm{d}}{\rm{o}}{\rm{s}}{\rm{e}}}}\times 100,$$

and

2 $${A}_{{\rm{s}}{\rm{o}}{\rm{u}}{\rm{r}}{\rm{c}}{\rm{e}}{\rm{o}}{\rm{r}}{\rm{g}}{\rm{a}}{\rm{n}}}\,(t)=cp{m}_{{\rm{s}}{\rm{o}}{\rm{u}}{\rm{r}}{\rm{c}}{\rm{e}}{\rm{o}}{\rm{r}}{\rm{g}}{\rm{a}}{\rm{n}}}\,(t)\times \frac{{A}_{{\rm{r}}{\rm{e}}{\rm{f}}{\rm{e}}{\rm{r}}{\rm{e}}{\rm{n}}{\rm{c}}{\rm{e}}{\rm{s}}{\rm{o}}{\rm{u}}{\rm{r}}{\rm{c}}{\rm{e}}}\times {e}^{-({\rm{l}}{\rm{n}}2/{T}_{1/2})\cdot t}}{cp{m}_{{\rm{r}}{\rm{e}}{\rm{f}}{\rm{e}}{\rm{r}}{\rm{e}}{\rm{n}}{\rm{c}}{\rm{e}}{\rm{s}}{\rm{o}}{\rm{u}}{\rm{r}}{\rm{c}}{\rm{e}}}\,(t)},$$

where ${\rm{ \% }}I{D}_{{\rm{s}}{\rm{o}}{\rm{u}}{\rm{r}}{\rm{c}}{\rm{e}}{\rm{o}}{\rm{r}}{\rm{g}}{\rm{a}}{\rm{n}}}\,(t)$ represents the *%ID* of the source organ measured at time *t* after injection; ${A}_{{\rm{s}}{\rm{o}}{\rm{u}}{\rm{r}}{\rm{c}}{\rm{e}}{\rm{o}}{\rm{r}}{\rm{g}}{\rm{a}}{\rm{n}}}\,(t)$, ${A}_{{\rm{i}}{\rm{n}}{\rm{j}}{\rm{e}}{\rm{c}}{\rm{t}}{\rm{e}}{\rm{d}}{\rm{d}}{\rm{o}}{\rm{s}}{\rm{e}}}$ and ${A}_{{\rm{r}}{\rm{e}}{\rm{f}}{\rm{e}}{\rm{r}}{\rm{e}}{\rm{n}}{\rm{c}}{\rm{e}}{\rm{s}}{\rm{o}}{\rm{u}}{\rm{r}}{\rm{c}}{\rm{e}}}$ represent the activity of the source organ at time *t* after injection, the injected dose, and the activity of the reference source at the injection time (the time of the prepared reference source is as the same with the injection time), respectively; $cpm(t)$ represents the counts per minute (cpm) of the reference source or source organs at time *t*; and ${T}_{1/2}$ is the physical half-life of the nuclide ^99m^Tc.

$cp{m}_{{\rm{s}}{\rm{o}}{\rm{u}}{\rm{r}}{\rm{c}}{\rm{e}}{\rm{o}}{\rm{r}}{\rm{g}}{\rm{a}}{\rm{n}}}\,(t)$ and $cp{m}_{{\rm{r}}{\rm{e}}{\rm{f}}{\rm{e}}{\rm{r}}{\rm{e}}{\rm{n}}{\rm{c}}{\rm{e}}{\rm{s}}{\rm{o}}{\rm{u}}{\rm{r}}{\rm{c}}{\rm{e}}}\,(t)$ were extrapolated from the projected images at each time point of 0.5 h, 1 h, 2 h, 4 h and 8 h. In general, the measured kinetic data (with the exclusion of that for the urinary bladder) could be represented as mathematical expression with one or more exponential terms, and the OLINDA/EXM 1.1 code (Vanderbilt University, Nashville, TN, USA) allowed the user to enter kinetic data for each source organ (*%ID* at different times) and fit it to one or more exponential terms^22^. The time-${\rm{ \% }}I{D}_{{\rm{s}}{\rm{o}}{\rm{u}}{\rm{r}}{\rm{c}}{\rm{e}}{\rm{o}}{\rm{r}}{\rm{g}}{\rm{a}}{\rm{n}}}\,(t)$ curves were fitted by least-squares analysis using the EXM module in the OLINDA/EXM 1.1 application. The areas under the fitted time-${\rm{ \% }}I{D}_{{\rm{s}}{\rm{o}}{\rm{u}}{\rm{r}}{\rm{c}}{\rm{e}}{\rm{o}}{\rm{r}}{\rm{g}}{\rm{a}}{\rm{n}}}\,(t)$ curves, which represent the TIACs, were calculated based on the physical ${T}_{1/2}$ of the radioisotope and the integral of the fitted time-activity curve from a time of zero to infinity. In this study, the curves for the ^99m^Tc-HYNIC-PSMA time-*%ID* data for the source organs was fitted with a single exponential equation $ID \% (t)=C\cdot \exp (\,-\,ct)$, and the TIAC was given by the integration of the curve, the result of which was C/c.

The total urine excretion $U{E}_{8h}$ at 8 h after the tracer injection was estimated from the acquired images as follows (Eqn. (3)):

3 $$U{E}_{8h}={A}_{0}-{A}_{WB-8h}^{{\rm{{\prime} }}}$$

where ${A}_{WB-8h}^{{\rm{{\prime} }}}$ is the whole body activity corrected according to the decay at 8 h after the administration and ${A}_{0}$ is the injection activity. The 2-phase exponential association curve was then fitted to the cumulative urine activity using the following formula (Eqn. (4)):

4 $$U(t)=U{E}_{8h}\times [1-A\exp \,(\,-\,\mathrm{ln}\,2\times t/{T}_{1/2}^{A})-B\exp (\,-\,\mathrm{ln}\,2\times t/{T}_{1/2}^{B})],$$

where $U(t)$ is the accumulated urine at time *t*, A and B are regression parameters, ${T}_{1/2}^{A}$is the A phase half-life and ${T}_{1/2}^{B}$ is the B phase half-life. The fraction of the tracer excreted through the bladder and the corresponding ${T}_{1/2}$ value were assessed according to the accumulated excreted activity calculated according to the whole body time-*%ID* curve.

To assess the TIAC of urinary bladder content, $U{E}_{8h}/{A}_{0}\cdot [A/(A+B)]$, $U{E}_{8h}/{A}_{0}\cdot [B/(A+B)]$, ${T}_{1/2}^{A}$and ${T}_{1/2}^{B}$ were used in the bladder-voiding model in OLINDA/EXM 1.1, with a voiding interval of 2 h. The TIAC of the rest of the body was obtained by the following formula (Eqn. (5)):

5 $$TIA{C}_{{\rm{rest}}}=TIA{C}_{{\rm{WB}}}-\sum _{source}TIA{C}_{source}-TIA{C}_{{\rm{bladder}}}$$

where $TIA{C}_{{\rm{rest}}}$ is the TIAC of the rest of the tissue, $TIA{C}_{{\rm{WB}}}$ is the TIAC of whole body, $TIA{C}_{source}$ is the TIAC of the source organs (lungs, kidneys, liver, spleen, salivary glands and heart) and $TIA{C}_{{\rm{bladder}}}$ is the TIAC of the urinary bladder.

The absorbed doses were estimated using the organ TIAC data described above, by the OLINDA/EXM 1.1 program^22–24^. The absorbed dose of the salivary glands was calculated based on the mass and S-values obtained from Liu *et al*.^25^. The effective dose (ED) is the sum of the weighted doses of each of the organs, which were calculated by multiplying the absorbed doses for the individual organ doses by a stochastic risk weighting factor (ICRP 103). The radiation transport phantom selected from OLINDA/EXM 1.1 was the hermaphroditic phantom, which is defined by Cristy and Eckerman as a 73.7 kg adult phantom.
