# Author Correction: Evaluation of Radiation dosimetry of ^99m^Tc-HYNIC-PSMA and imaging in prostate cancer

**DOI:** 10.1038/s41598-020-74851-x

**Published:** 2020-10-23

**Authors:** Jianping Zhang, Jiangang Zhang, Xiaoping Xu, Linjun Lu, Silong Hu, Chang Liu, Jingyi Cheng, Shaoli Song, Yingjian Zhang, L. Q. Shi

**Affiliations:** 1 Key Laboratory of Nuclear Physics and Ion-Beam Application (MOE), Fudan University, No. 220, Handan Road, Yangpu District, Shanghai, 200433 China; 2 Institute of Modern Physics, Fudan University, No. 220, Handan Road, Yangpu District, Shanghai, 200433 China; 3 Department of Nuclear Medicine, Fudan University Shanghai Cancer Center, No. 270, Dong’an Road, Xuhui District, Shanghai, 200032 China; 4 Department of Oncology, Shanghai Medical College, Fudan University, No. 130, Dong’an Road, Xuhui District, Shanghai, 200032 China; 5 Center for Biomedical Imaging, Fudan University, No. 270, Dong’an Road, Shanghai, 200032 China; 6 Shanghai Engineering Research Center for Molecular Imaging Probes, No. 270, Dong’an Road, Xuhui District, Shanghai, 200032 China; 7 Department of Nuclear Medicine, Shanghai Proton and Heavy Ion Center, Fudan University Cancer Hospital, No. 4365, Kangxin Road, Pudong New District, Shanghai, 201315 China; 8 Shanghai Engineering Research Center of Proton and Heavy Ion Radiation Therapy, No. 4365, Kangxin Road, Pudong New District, Shanghai, 201315 China

Correction to: *Scientific Reports* 10.1038/s41598-020-61129-5, published online 06 March 2020

This Article contains a typographical error in the Acknowledgements section.

“The authors would like to thank GE Healthcare China for technical assistance. This study was carried out with financial support from the General Project of the Shanghai Municipal Health and Family Planning Commission Foundation, China (Grant No. 20170425) and Shanghai Engineering Research Center of Molecular Imaging Probes, China (Grant No. 19DZ2282200).”

should read:

“The authors would like to thank GE Healthcare China for technical assistance. This study was carried out with financial support from the General Project of the Shanghai Municipal Health and Family Planning Commission Foundation, China (Grant No. 201740185) and Shanghai Engineering Research Center of Molecular Imaging Probes, China (Grant No. 19DZ2282200).”

